# When AI Meets *Pakikiramdam*: Humanizing Digital Health in Philippine Family Practice

**DOI:** 10.1002/jgf2.70078

**Published:** 2025-10-15

**Authors:** Ivan Efreaim A. Gozum

**Affiliations:** ^1^ University of Santo Tomas Manila Philippines

To the Editor,

1

In a study by Safdar and Aslam, they have recommended that future directions for chronic kidney disease in the primary care setting can emphasize personalized medicine, leveraging biomarkers and artificial intelligence (AI) to predict disease progression and tailor treatments [1]. With this, I would like to discuss the suggested appropriate integration of artificial intelligence in family care. According to research, one of the most significant and possible sectors that will continue to thrive is medicine and healthcare, which involves prediction, management, and healing [2]. For this reason, it can be noted how the growing integration of AI and digital technologies into clinical care is reshaping family medicine worldwide. For example, AI can be used for detecting potential cases of depression for those who are undergoing care so that preventive measures and potential cures can already be prepared for patients.

In the early stages, in the Philippines, AI tools, from automated triage to predictive diagnostics, are increasingly seen as solutions to healthcare gaps, especially in under‐resourced settings [3]. However, as promising as these technologies are, they pose critical ethical and cultural challenges. The risks that AI potentially engenders do not only concern inequality; rather, it asks healthcare professionals to rethink the way healthcare can utilize AI as a tool. In particular, they risk displacing essential Filipino values such as *pakikiramdam*—a culturally embedded form of empathetic attunement that is central to patient‐physician relationships [4].

AI excels at processing data, but it does not “feel” the nuanced rhythms of *kapwa* (shared identity) or the silences and subtleties that Filipino physicians often navigate in the clinic. In family medicine, where relational trust and unspoken understanding often guide care decisions, the mechanization of care can flatten human presence into mere algorithmic recommendations. Therefore, *pakikiramdam* is not just cultural; it reflects a deeper theological anthropology, where each person is seen not merely as a unit of health data but as *imago Dei*, created for relationship and care.

The risk, then, is that clinical decision‐making becomes overly technocratic, sidelining the relational and spiritual aspects of healing. In a society where health is inherently communal and moral discernment is often shared by the family and the barangay, AI systems must be designed and deployed with contextual sensitivity. Digital tools must be made to serve, and not override, the practices of listening, presence, and moral accompaniment that define Filipino family medicine. At the end of the day, a true *pakikiramdam* requires a healthcare system that is more human and goes beyond the digital screens that AI is leaning towards [5].

The solution is not to reject technology but to embed human values into it, particularly the values rooted in our culture and faith. This involves interdisciplinary collaboration between physicians, ethicists, and AI developers. As family medicine evolves in the digital age, let us ensure that it remains a space where technology enhances, rather than replaces, relational care. Informed by *pakikiramdam*, and the lived wisdom of Filipino communities, we can develop a vision of digital health that is both innovative and deeply human.

## Author Contributions

The whole article is written by Ivan Efreaim A. Gozum.

## Conflicts of Interest

The author declares no conflicts of interest.

## Linked Articles

This article is linked to https://doi.org/10.1002/jgf2.70054.

## Data Availability

The author has nothing to report.
